# Developmental and tissue specific changes of ubiquitin forms in *Drosophila melanogaster*

**DOI:** 10.1371/journal.pone.0209080

**Published:** 2018-12-13

**Authors:** Ágota Nagy, Levente Kovács, Zoltán Lipinszki, Margit Pál, Péter Deák

**Affiliations:** 1 Department of Genetics, University of Szeged, Szeged, Hungary; 2 Institute of Biochemistry, Biological Research Centre, Szeged, Hungary; 3 MTA SZBK Lendület Laboratory of Cell Cycle Regulation, Biological Research Centre, Szeged, Hungary; CHA University, REPUBLIC OF KOREA

## Abstract

In most Eukaryotes, ubiquitin either exists as free monoubiquitin or as a molecule that is covalently linked to other proteins. These two forms cycle between each other and due to the concerted antagonistic activity of ubiquitylating and deubiquitylating enzymes, an intracellular ubiquitin equilibrium is maintained that is essential for normal biological function. However, measuring the level and ratio of these forms of ubiquitin has been difficult and time consuming. In this paper, we have adapted a simple immunoblotting technique to monitor ubiquitin content and equilibrium dynamics in different developmental stages and tissues of *Drosophila*. Our data show that the level of total ubiquitin is distinct in different developmental stages, lowest at the larval-pupal transition and in three days old adult males, and highest in first instar larvae. Interestingly, the ratio of free mono-ubiquitin remains within 30–50% range of the total throughout larval development, but peaks to 70–80% at the larval-pupal and the pupal-adult transitions. It stays within the 70–80% range in adults. In developmentally and physiologically active tissues, the ratio of free ubiquitin is similarly high, most likely reflecting a high demand for ubiquitin availability. We also used this method to demonstrate the disruption of the finely tuned ubiquitin equilibrium by the abolition of proteasome function or the housekeeping deubiquitylase, Usp5. Our data support the notion that the ubiquitin equilibrium is regulated by tissue- and developmental stage-specific mechanisms.

## Introduction

Ubiquitin (Ub) is an evolutionarily conserved short polypeptide of 76 amino acids. As a post-translational protein modifier, it plays a critical role in many intracellular processes. Ubiquitin modification occurs through the process of ubiquitylation, in which single or multiple ubiquitin moieties bind covalently to target proteins [1]. This process is regulated by ubiquitin ligases. Such ubiquitin attachments are reversed in the process of deubiquitylation by deubiquitylating (DUB) enzymes. Due to the reversible nature of this modification, the ubiquitin pool of cells is divided into distinct fractions that include free monoubiquitins as well as covalently linked mono- and polyubiquitin-protein conjugates and unanchored polyubiquitin chains. These ubiquitin forms reach a dynamic intracellular equilibrium in which the availability of free monoubiquitins appears to be essential for normal cell physiology. Multiple regulatory mechanisms have been described that ensure physiologically required ubiquitin levels are maintained. These include transcriptional regulation of ubiquitin coding genes, the regulation of ubiquitin recycling from conjugated or free polyubiquitins, and ubiquitin degradation [2]. For example, it was shown that certain ubiquitin-coding genes are transcriptionally activated during different stress conditions [3, 4] when more monoubiquitins are required for the ubiquitylation and degradation of an excess of misfolded proteins. Similarly, the free monoubiquitin pool could be boosted by regulation of DUB expression and activity [5,6] that liberates free ubiquitin from their conjugated forms upon stress induction.

Precise measurement of the ubiquitin pool and the ratio of free versus conjugated ubiquitin forms, or their cycle dynamics, is an important step in the study of the ubiquitylation machinery and to better understand ubiquitin regulated intracellular processes. The first quantitative assays (including RIA, solid-phase EIA and ELISA) were able to determine the two pools of ubiquitin from cell homogenates by using antibodies that discriminate between free and conjugated forms [7,8,9]. While these were relatively simple methods, their accuracy was doubtful, because the specificity of the antibodies used were not exclusive to one form of ubiquitin only. A Usp2 coupled indirect competitive ELISA assay overcame the selectivity problem associated with the Ub-specific antibodies. In this assay, all conjugated ubiquitins in a biological sample are converted to free monoubiquitins by the Ub-specific protease, Usp2, then quantified using Ub-ELISA [10]. Although it is a simple and reliable test, in its original form it is restricted only for total ubiquitin determination. Since then, a mass spectrometry (MS) based technique—ubiquitin protein standard absolute quantification (Ub-PSAQ)—has been established that combines differential affinity chromatography with MS [11]. While this is the most accurate measurement to quantify different ubiquitin forms in cell homogenates, it is admittedly the most complicated, expensive and time-consuming. Moreover, it requires a mass spectrometry facility, therefore it is not an obvious choice for large scale screens and phenotypic characterisations.

Recently, a simple and elegant immunoassay was developed for simultaneous determination of total (Ub_T_), as well as free (Ub_F_) and conjugated (Ub_C_) ubiquitins from mouse whole protein extracts by densitometric analysis of western blots [12]. In this assay, similarly to the Usp2-based assay mentioned above, the Ub_T_ content of cell lysates is determined in the form of monoubiquitins. However, instead of adding external Usp2 activity, endogenous DUBs present in the sample homogenate after lyses process all conjugated ubiquitins to monoubiquitins. The Ub_F_ fraction is in turn determined from similar lysates supplemented with strong DUB inhibitors. Appropriate samples of these lysates are immunoblotted along with Ub standards that allow the quantification of Ub_T_ and the Ub_F_ fraction by densitometric analysis; as well as the calculation of Ub_C_ by subtracting Ub_F_ from Ub_T_. After adapting this assay to *Drosophila*, we determined the Ub_T_, Ub_F_ and Ub_C_ concentrations in different critical developmental stages, in various physiologically active tissues of *Drosophila melanogaster*, and in mutants disrupting either proteasome function or Ub recycling. Our data demonstrate for the first time the highly dynamic nature of ubiquitin equilibrium throughout development of a multicellular organism.

## Materials and methods

### *Drosophila* stocks

An isogenized *Drosophila melanogaster OregonR* strain was used in these experiments. The flies were maintained in standard *Drosophila* medium at 25°C. The *Usp5*^*RNAi*^ line was obtained from the Bloomington *Drosophila* Stock Center (Stock number: 31886). To generate the transgenic Rpn10/p54 RNAi line (designated as *p54*^*RNAi*^ in this paper), PCR was performed with primer pairs of 5’-attctagacgttgaagtgctggccactc-3’ (Fw) and 5’-gctctagatgttggcttcgttctctgct-3’ (Rev) to amplify a 656 bps sequence corresponding to nucleotides 171–826 of the CDS (Flybase ID: CG7619-PA) of Rpn10/p54. The PCR fragment was directly cloned into the pWIZ vector, in opposite orientations on both sides of the white intron (in 3’–5’-intron-5’–3’configuration) according to Lee & Carthew [13]. The Rpn10/p54-specific RNAi construct was designated as pP{UAST-p54^RNAi^} and verified by DNA sequencing before embryo (*w*^*1118*^) injection. Transgenic flies were generated by random P-element insertion following standard procedures. The UAS-Gal4 system [14], with the daughterless-Gal4 driver, was used to induce dsRNA expression from the transgene.

### Sample preparations

For the preparation of whole protein extracts, approximately 5–6 mg of synchronized samples were collected from different developmental stages, chilled and kept frozen at -80°C. Tissue samples were dissected from third stage larvae (larval brain, imaginal discs, salivary glands, fat body) and three day old adults (testes, ovaries and heads), chilled and kept frozen at -80°C. A minimum three independent samples were prepared from each developmental stage, tissue or mutant. The animal and tissue samples were homogenized by plastic tissue grinders in pre-chilled microfuge tubes either in 100 μl ice cold buffer F [100 mM Tris, pH 7.6, 150 mM NaCl, 1mM EDTA, 10 mM N-Ethylmaleimide (NEM, Sigma-Aldrich), 20μM MG132 (Calbiochem) and 1× EDTA-Free Complete Protease Inhibitor Cocktail (Roche)] or 100 μl buffer T [Buffer F supplemented with 2mM DTT to preserve the catalytic activity of DUBs, but without NEM], respectively. Following centrifugation (4°C, 10 minutes, 21000 g), 60 μl supernatants (whole protein extracts) were collected for further analysis. Total protein content of the supernatants was determined by the Qubit Protein Assay Kit (Thermo Fisher Scientific Inc.) according to the manufacturer’s protocol using a Qubit 2 benchtop fluorometer (Invitrogen). The kit is based on the NanoOrange Protein Quantitation Assay [15]. Protein extracts in buffer F were supplemented with 4x Laemmli sample buffer and heat inactivated by boiling for 3 minutes. Samples made in buffer T were incubated at 25°C for 3 hours, and then mixed with 4x Laemmli sample buffer (20 μl sample buffer to 60 μl sample) and boiled for 3 minutes.

### Western blot analysis

Appropriate amounts (based on protein concentration) of protein extracts in sample buffer were loaded onto 14%—home-casted—1 mm thick Tris-Glycine SDS-PAGE gels. Two technical repeats from all samples were loaded to each gel. Separation was performed at a constant 150 V in a buffer containing 25 mM Tris pH 8.3 and 192 mM Glycine. On each gel, 0.5, 1, 2 and 3 pmol ubiquitin (Sigma-Aldrich) samples were run as standards. Following separation, proteins were blotted onto a PVDF membrane (Merck Immobilon-P) by standard wet transfer (3h, 300 mA constant current, in transfer buffer containing 20 mM Tris pH 8.0, 150 mM Glycine and 20% Methanol). Membranes were blocked in 5% non-fat milk (in TBS (10mM Tris pH 8.0, 150 mM NaCl)) for 30 minutes at room temperature, then incubated with polyclonal anti-Ub antibody (rabbit, Dako) diluted 1:1000 in TBST+B (TBS supplemented with 0.05% Tween-20 and 1% bovine serum albumin) for 1 hour at room temperature. After washing three times in TBST (10 minutes each), the membranes were incubated with horseradish peroxidase (HRP) conjugated goat anti-rabbit secondary antibody diluted 1:30 000 in TBST+B for 1 hour at room temperature, followed by washing in TBST (3 times, 10 min each). Membranes were covered with Saran Wrap and ECL reaction was performed in the dark using Immobilon Western Chemiluminescent HRP Substrate (Merck), X-ray films (Fujifilm) and Tetenal X-ray film processing developer and fixative solutions.

### Monoubiquitin quantification

The developed X-ray films were digitalized with a BioDoc-It Imaging System (UVP) using a white light conversion adapter. The monoubiquitin content of the samples were determined by densitometric analysis of bands corresponding to the Ub standards and the samples using the ImageJ software (NIH, Bethesda, Maryland). Calibration curves were generated for each western blot by plotting band intensities against Ub standard concentrations in MS Excel XLSTAT. A regression line equation was generated by applying the four parameter curve fit model [16], which best suits the sigmoid distribution of the data. It is then used to calculate the Ub concentrations in the developmental and tissue samples that were normalized to total protein content. In our assays, the coefficient of determination (R^2^) was always between 0.99–1. Intra- and inter-assay variation coefficient (as a percentage, CV%) was calculated to assess reproducibility by dividing the standard deviation of the mean by the mean value, then multiplying by 100. Intra-assay CV% was determined for identical sample duplicates and triplicates, while inter-assay CV% was calculated for sample triplicates analyzed for each time point or tissue [17]. For multiple comparison of the developmental stage-specific and tissue samples, one-way ANOVA was performed followed by an SNK (Sudet-Newman-Keuls) post-hoc test (S1 Table). The mutant samples were tested using Welch’s t test. R statistical software was used for all statistical computing.

## Results

### Assay conditions

A simple immunoblot-based assay was originally developed for ubiquitin quantification in mouse tissue lysates [12], thus we first needed to adapt it to *Drosophila*. The optimal composition, pH and ion concentration of the homogenization buffers, as well as the optimal incubation temperature, were determined experimentally and adjusted accordingly (see S3 Table). To preserve conjugated ubiquitins for Ub_F_ measurements, we used EDTA, a complete protease inhibitor cocktail and N-Ethylmaleimide (NEM) in buffer F to inhibit DUBs with metallo-, serine- and cysteine protease activities. We preferred to use NEM over IAA (Iodoacetamide) to alkylate, and therefore inactivate the active site cysteine residues of DUBs, because it is more stable than IAA, less sensitive to light and does not interfere with downstream applications [18]. The free monoubiquitin fraction of *Drosophila* whole protein extracts were separated from the conjugated ubiquitin forms by SDS-PAGE coupled to western blotting as a single Ub-immunoreactive band at about 8.5 kDa. This fraction includes, in addition to the free ubiquitins, the activated ubiquitin-adenylate and the thioester bond ubiquitin intermediates, because these are inherently unstable, and dissociate to free monoubiquitins at the protein extraction conditions [19]. For total ubiquitin determination, the homogenates were incubated at room temperature in buffer T that lacked DUB inhibitors.

**Fig 1:**
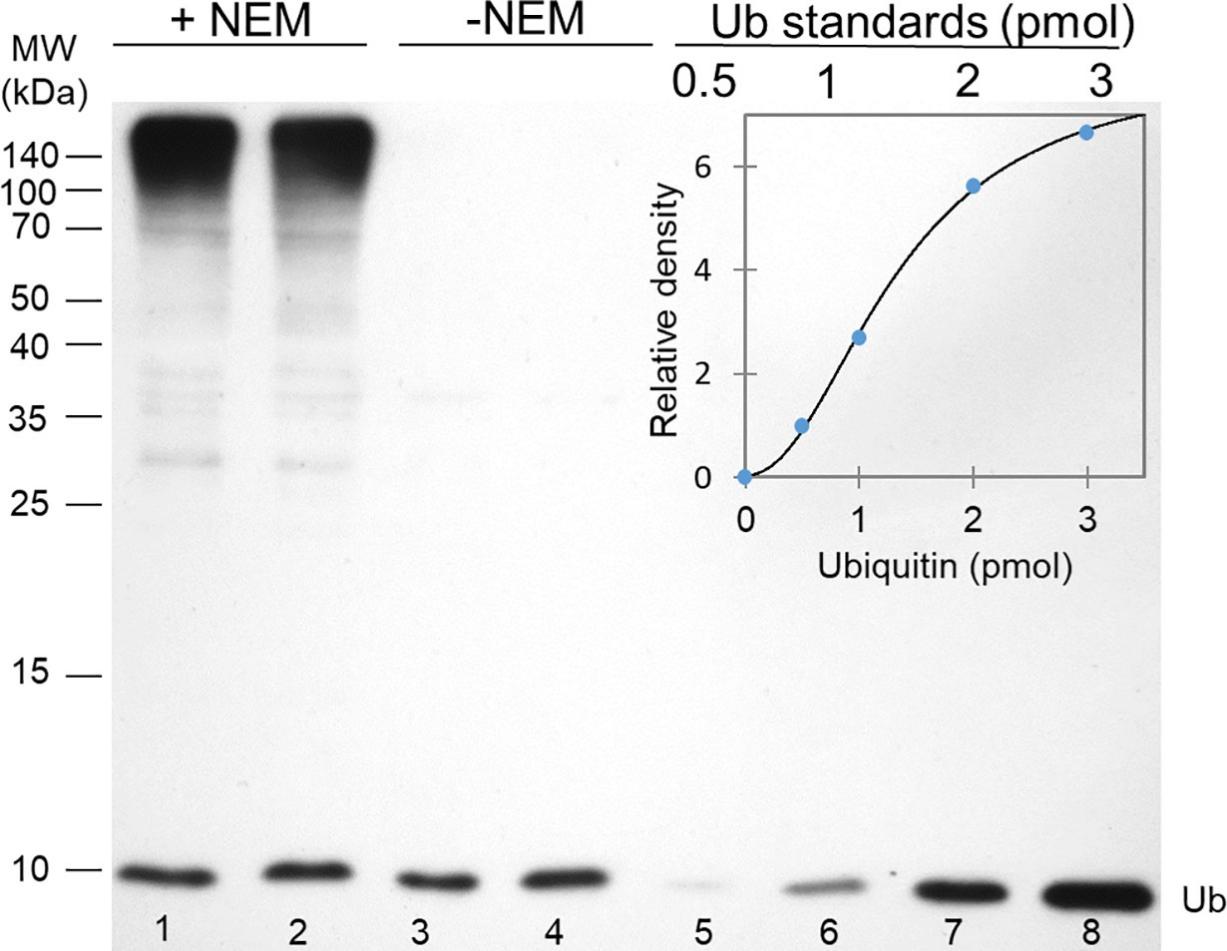
An immunoblot based assay to quantitate free and total ubiquitin content of whole protein extracts. In this blot, parallel pupal samples (from stage P4 pupae) homogenized in buffers F (lanes 1 and 2; +NEM) and buffer T (lanes 3 and 4; -NEM) were loaded and polyclonal anti-Ub antibody at a dilution of 1:1000 was used to detect ubiquitin. Only the intensity of the free monoubiquitin band (just below the 10 kDa mark) was determined and used for quantification. 5 μg total protein extracts were loaded to lanes 1 and 2, while samples were diluted twofold before loading to lanes 3 and 4 to avoid overloading the monoubiquitin band. Ub standards of 0.5, 1, 2 and 3 pmol were loaded to lane 5–8 respectively, for the calibration curve. The inset shows the calibration curve that was used to calculate Ub concentrations in these samples. Band intensities were plotted against Ub standards and a regression line equation was generated by applying the four parameter curve fit model (R^2^ = 0.9978).

A 3-hour incubation time was sufficient to convert the conjugated ubiquitins to free mono-ubiquitins by the endogenous DUBs. Lanes 3 and 4 in Fig 1 demonstrates that essentially there is no Ub-immunoreactive signal detectable outside the monoubiquitin band on a western blot. Ub standards of the 0.5–3 pmol range were run on each SDS-PAGE gel for creating calibration curves, which were then used for quantification of the monoubiquitin content (normalized to total protein content) of the samples. In this range, band intensity data were neatly following a sigmoidal shaped curve. (Fig 1) with high correlation coefficients. The precision and reproducibility of the quantification were estimated from intra- and inter-assay variations, which were below 10% and 18%, respectively.

Although ubiquitin is considered as a stable polypeptide, and a complete protease inhibitor cocktail was added to each of the protein extracts, changes in the total ubiquitin pool in wandering L3 larval samples were monitored up to 5 hours of incubation in buffer T at 25 degrees. The results of this experiment are summarized in S4 Table and show that the total Ub pool remains the same even after 5 hours of incubation.

### Ubiquitin pool dynamics during development

**Fig 2:**
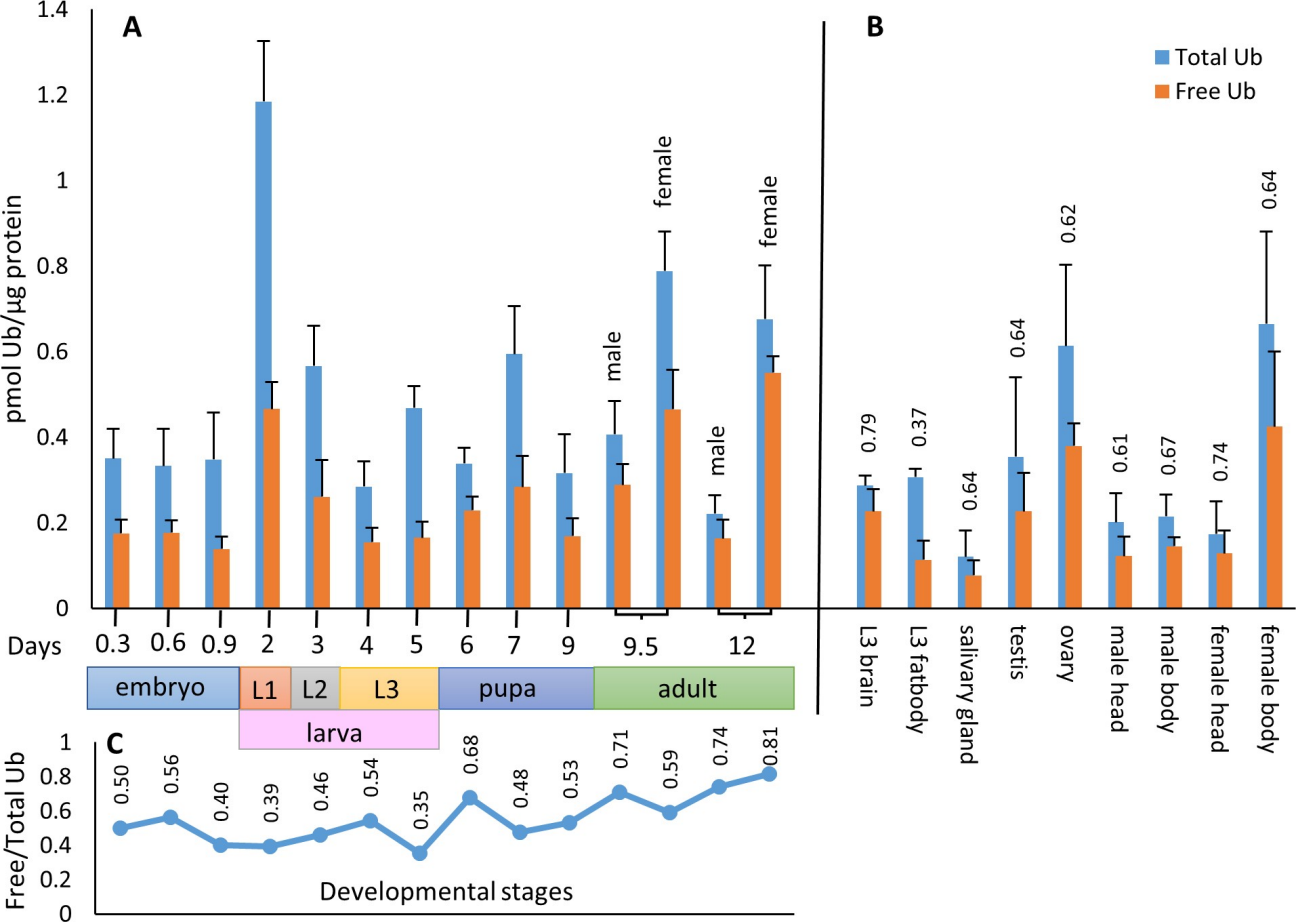
**Developmental (A) and tissue specific (B) profile of free (light blue) and total (dark blue) ubiquitins and free/total ubiquitin ratios (C and above columns in B).** The colour bar at the bottom indicates the length of the developmental stages at 25°C. For panel B, tissues were prepared from third instar larvae (L3) and three day old adults. Ubiquitin content was normalized to total protein content. Data are presented as mean±SD (see also S1 Table) of three independent experiments (n = 3). The data were compared using one-way analysis of variance (ANOVA) followed by SNK post-hoc test (see S2 Table).

To quantitate cellular ubiquitin pool components by this assay, whole protein extracts were prepared in Buffers F and T respectively, from all stages of *Drosophila* development, and analyzed together with Ub standards on western blots using a rabbit polyclonal anti-Ub antibody. Ub concentrations were determined by densitometric analysis of the band intensities of Ub standards and the samples. Fig 2A shows the developmental profile consistently obtained for Ub_T_ and the Ub_F_ fractions. The total Ub content appears to be constant in embryos, then peaks in L1, and gradually decreases in L2 and L3 stages. It climbs again at the larval-pupal transition and during pupal development (metamorphosis) and reaches a relatively high value in adults, which is more pronounced in females than in males. Interestingly, the ratio of Ub_F_ stays between 30–50% of total Ub during larval and pupal development, but climbs to 70–80% at the larval-pupal and the pupal-adult transitions; then it stays within that range in the adults (Fig 2C).

### Ubiquitin pool distribution in larval and adult tissues

We have also applied this immunoblot-based assay to determine the composition of the ubiquitin pool in larval tissues, including the mitotically active larval brains, salivary glands and fat bodies; as well as in adult body parts, such as head, ovary and testis. Total ubiquitin levels are quite low in the adult CNS (in male and female heads), higher in testes and relatively high in the ovaries (Fig 2B). Therefore, it is most likely that the high levels of ubiquitin found in adult females originates mainly from the ovaries. This suggests that ubiquitin-dependent processes may play a key role in the reproduction of animals. It would be interesting to examine whether this is related to mitosis and meiosis, since many events in the cell cycle depend on protein ubiquitination and degradation. Fig 2B also illustrates that mitotically or developmentally active tissues [20], such as larval brain, testis, ovary, and the CNS contain the highest percentage of free monoubiquitins; while it is the lowest in larval fat bodies and salivary glands. These data indicate that the normal functioning of the CNS in larvae and adults, as well as testes and ovaries, may require high proportion of free monoubiquitins.

### Changes in ubiquitin forms via loss of Rpn10/p54 and Usp5 function

Ubiquitin equilibrium is regulated, among other factors, by deubiquitinating enzymes and normal functioning of the proteasome. It has been shown that the loss-of-function mutation in the Rpn10/p54 polyubiquitin receptor subunit of the proteasome [21,22], and in one of the DUB genes in *Drosophila*, Usp5 [23,24], leads to disruption of ubiquitin equilibrium and an excessive accumulation of free polyubiquitins and polyubiquitylated proteins. To quantitate these changes, we applied the immunoblot-based ubiquitin quantification assay to determine the steady-state distribution of ubiquitin forms in animals with impaired Rpn10/p54 and Usp5 functions and compared them to wild type data.

**Fig 3:**
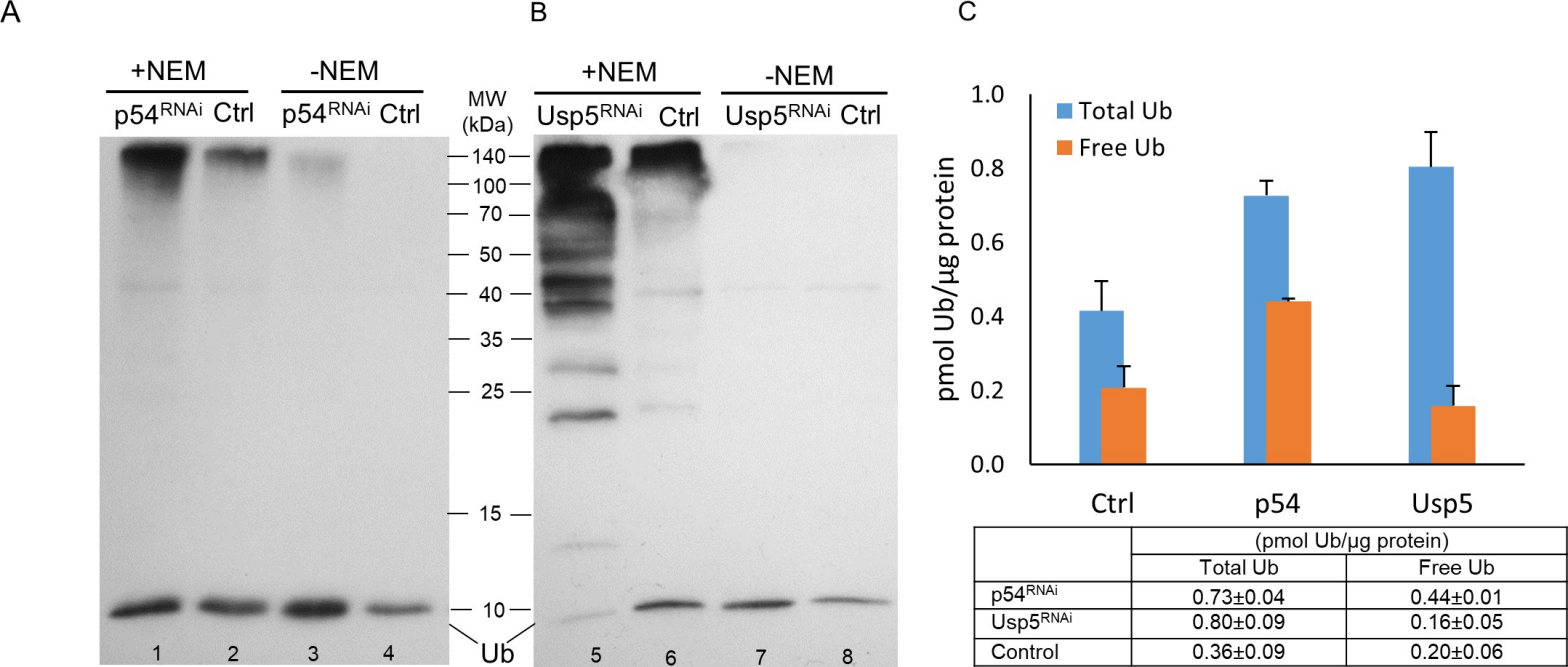
**Effect of loss of Rpn10/p54 (A and C) proteasome subunit or Usp5 deubiquitylase (B and C) on the abundance of ubiquitin forms.** Whole protein extracts in buffer F (lanes 1, 2, 5 and 6; +NEM) and buffer T (lanes 3, 4, 7 and 8; -NEM) of wandering L3 larvae were investigated by western blotting using polyclonal anti-Ub antibody at a dilution of 1:1000. The bands just below the 10 kDa mark on the immunoblots (A and B) represent free monoubiquitins, and only the intensity of these bands were determined and used for quantification. 5 μg total protein extracts were loaded to lanes 2 and 6, while samples were diluted 1.7-fold to lanes 1 and 5; twofold to lanes 3, 4 and 8; and 3.3-fold to lane 7 before loading to avoid overloading the monoubiquitin band. Ubiquitin content (small table in C) was calculated by plotting band intensities against Ub standards and a regression line equation was generated by applying the four parameter curve fit model (R^2^ = 0.9979 for Rpn10/p54 and R^2^ = 0.9933 for Usp5), values were normalized to total protein content and shown as a column diagram.

Disrupting the function of either the proteasome subunit, or a deubiquitylase, results in about a twofold increase in total ubiquitins, but with significantly different characteristics. As Fig 3A and 3C show, in *Rpn10/p54* RNAi-silenced animals, an almost twofold increase in total ubiquitin (p-value = 6.523e-07 using Welch’s t-test) went hand in hand with about a twofold increase in free monoubiquitins (p-value = 1.387e-08). In contrast, *Usp5* silencing is concomitant with a robust increase in the abundance of free polyubiquitin chains (Fig 3B, ladder-like protein bands) that is reflected in a twofold increase in total ubiquitins (Fig 3C, p value = 0.009568) and a slight, not significant, reduction of free monoubiquitins (Fig 3B and 3C, p-value = 0.2581). These phenotypic differences in ubiquitin pool dynamics might represent unique responses to different perturbations of the ubiquitin equilibrium.

It should be noted that the total ubiquitin content of the animals with RNAi silenced *Rpn10/p54* gene, must be higher than the value given in our assay; since even after 3 hours of incubation, we detected some amount of conjugated ubiquitin species that remained in the cell lysates. This is evident from the high molecular weight section of Fig 3A (Lane 3). A possible explanation for this phenomenon may be a partial downregulation of the deubiquitinating enzymes responsible for ubiquitin deconjugation in the *Rpn10/p54* mutant cells due to weakened association of some DUBs with the proteasome [25,26]. In any case, these data indicate that the immunoblot-based ubiquitin quantification assay can be used to detect dynamic changes in the ubiquitin pool.

## Discussion

Ubiquitin regulates almost all intracellular processes through its reversible conjugation to substrate proteins. The intracellular ubiquitin pool consists of free and conjugated ubiquitins and a dynamic equilibrium is maintained between these forms that ensures the availability of free ubiquitins for ubiquitin-dependent cellular processes. Genetic dissection of ubiquitin-dependent cellular functions and the ubiquitylation—deubiquitylation machinery requires a simple and reliable method to monitor changes in ubiquitin pool dynamics. In this study, we adapted an experimental setup, developed by Oh et al. [12], for use in *Drosophila*. This assay was used to quantitate different ubiquitin forms throughout *Drosophila* development, in various tissues and mutants that interrupt the ubiquitylation machinery. The core element of the immunoblot-based ubiquitin quantification assay is densitometric analysis of western blot images. Multiple, parallel measurements can be done in animal and tissue homogenates with minimal pre-treatments within a 2-day period, and ubiquitin can be detected in the picomolar range. It is therefore quite fast and sensitive, and only requires equipment readily available in a molecular biology laboratory. Both intra- and inter-assay variation indicated acceptable reproducibility. This is achieved by using intra-assay standard curves for each immunoblot, with an appropriate range of ubiquitin standards.

One limitation of this method is that it cannot distinguish and quantitate all ubiquitin forms, such as free polyubiquitins, or mono- and polyubiquitylated proteins, since all of these become converted to monoubiquitins. Only free monoubiquitins and total ubiquitin levels can be determined with this assay, while covalently linked ubiquitins (*en bloc*) can only be calculated by subtracting the value for free ubiquitins from the total ubiquitin value.

Another limitation of the immunoblot-based ubiquitin quantification assay, in its present form, is that X-ray film is used for detection. While film-based detection is sensitive enough, its range of detection is quite narrow—about 4–8 fold [27]. It also tends to saturate rapidly when exposed to strong signals, making it difficult to accurately calculate the upper limit of detection. This limitation can be overcome by using NIR Western blots and a modern digital imaging apparatus (an RGB or NIR based CCD system) that offers a wider linear range of detection, detecting faint signals without saturating strong ones.

The developmental profile shown on Fig 2A indicates a highly dynamic nature of the ubiquitin pool, both in terms of the abundance of its components and their ratio. The most notable changes are the total ubiquitin peak in first instar larvae (L1), and the high free ubiquitin percentage at larval-pupal and pupal-adult transitions. These developmental transitions are characterized by complex remodeling of different tissues and abrupt proliferative activities. For example, many neuroblasts resume mitotic activity in first instar larvae to produce neurons that will make up most of the adult central and peripheral nervous systems [28]. It was shown that at least some of these reshaping processes, like pruning of axons and dendrites or the apoptosis of supernumerary neurons, are ubiquitin-mediated [29]. Although it is reasonable to think that similar ubiquitin-dependent processes operate in other tissues during development, further investigations are required to correlate these changes in the ubiquitin pool to molecular and cell biological events.

The loss of either Rpn10/p54 or Usp5 function led to quantifiable changes in the ubiquitin pool, although with visible differences (see Fig 3C). In the absence of the Rpn10/p54 polyubiquitin receptor, an increase in total ubiquitins can be explained by the lack of proteasome activity and consequently the cessation of proteasomal degradation of the ubiquitin itself [6,30]. Since the ubiquitin gene expression and recycling is unaffected, free monoubiquitins accumulate as well, and contribute to the increase in total ubiquitins. On the other hand, diminishing Usp5 ubiquitin carboxyl-terminal hydrolase function results in a considerable accumulation of free ubiquitin chains (Fig 3B) that likely abolish proteasomal activity by competitive inhibition [31, 32].This is a broader inhibition affecting most proteasomal substrates, hence the somewhat higher total ubiquitin value in these samples (Fig 3C). At the same time, the ubiquitin recycling is also disrupted in the absence of Usp5, which may explain the low level of free monoubiquitins.

In summary, we used an immunoblot-based ubiquitin quantification assay to detect and quantitate dynamic changes of ubiquitin pool components during *Drosophila* development, in several tissues and mutants. The simplicity of this assay makes it an excellent tool to study the effects of different factors on the ubiquitylation–deubiquitylation machinery or ubiquitin dependent intracellular functions.

## Supporting information

S1 Table Ubiquitin measurement data in *Drosophila* protein extracts. (DOCX)

S2 Table Multiple comparison of the sample means. One-way ANOVA was performed, followed by SNK (Student–Newman–Keuls, p < 0.05) post hoc test. Means indicated with the same letter are not significantly different.
(DOCX)

S3 Table Optimal conditions for the ubiquitin quantification assay. (DOCX)

S4 Table Ubiquitin stability in buffer T. After an initial 3-hour incubation, equal aliquots were taken in every 30 minutes that were subjected to SDS-PAGE followed by Western blotting. Relative ubiquitin content of the samples was determined by densitometric analysis of the immunoblots, and their Ub content was compared to the 3h samples in four independent experiments.
(DOCX)
