# Supplementary material for: Developmental and tissue specific changes of ubiquitin forms in Drosophila melanogaster
Source: PLoS One. 2018 Dec 13;13(12):e0209080. doi: 10.1371/journal.pone.0209080 (PMC6292614; doi:10.1371/journal.pone.0209080)
Supplement: S1 Table — (DOCX) [file pone.0209080.s001.docx]

| **Samples** | **Total Ubiquitin (UbT)** | | **Free Ubiquitin (UbF)** | | | **Free Ubiquitin/**  **Total ubiquitin**  **UbF/UbT** |
| --- | --- | --- | --- | --- | --- | --- |
|  | **Mean of concentrations**  **pmol Ub/µg total protein** | **Standard deviation**  **(SD)** | **Mean of concentrations**  **pmol Ub/µg total protein** | | **Standard deviation**  **(SD)** |  |
| **Developmental stages** | | | | | | |
| E0-3 | 0.35 | 0.07 | 0.18 | 0.03 | | 0.50 |
| E8-11 | 0.32 | 0.09 | 0.18 | 0.03 | | 0.56 |
| E16-19 | 0.35 | 0.11 | 0.14 | 0.03 | | 0.40 |
| L1 | 1.18 | 0.14 | 0.47 | 0.06 | | 0.39 |
| L2 | 0.57 | 0.09 | 0.26 | 0.09 | | 0.46 |
| eL3 | 0.28 | 0.06 | 0.15 | 0.03 | | 0.54 |
| vL3 | 0.47 | 0.05 | 0.17 | 0.04 | | 0.35 |
| P1 | 0.34 | 0.04 | 0.23 | 0.03 | | 0.68 |
| P4 | 0.59 | 0.11 | 0.28 | 0.07 | | 0.48 |
| P15 | 0.32 | 0.09 | 0.17 | 0.04 | | 0.53 |
| male0 | 0.41 | 0.08 | 0.29 | 0.05 | | 0.71 |
| male3 | 0.22 | 0.04 | 0.16 | 0.04 | | 0.74 |
| female0 | 0.79 | 0.09 | 0.46 | 0.09 | | 0.59 |
| female3 | 0.68 | 0.13 | 0.55 | 0.04 | | 0.81 |
| **Tissues** | | | | | | |
| L3 brain | 0.29 | 0.02 | 0.23 | 0.05 | | 0.79 |
| L3 fatbody | 0.31 | 0.02 | 0.11 | 0.04 | | 0.37 |
| salivary gland | 0.12 | 0.06 | 0.08 | 0.04 | | 0.64 |
| testis | 0.35 | 0.19 | 0.23 | 0.09 | | 0.64 |
| ovary | 0.61 | 0.19 | 0.38 | 0.05 | | 0.62 |
| male head | 0.20 | 0.07 | 0.12 | 0.05 | | 0.61 |
| male body | 0.21 | 0.05 | 0.14 | 0.02 | | 0.67 |
| female head | 0.17 | 0.08 | 0.13 | 0.05 | | 0.74 |
| female body | 0.66 | 0.22 | 0.42 | 0.18 | | 0.64 |
