# Supplementary material for: Developmental and tissue specific changes of ubiquitin forms in Drosophila melanogaster
Source: PLoS One. 2018 Dec 13;13(12):e0209080. doi: 10.1371/journal.pone.0209080 (PMC6292614; doi:10.1371/journal.pone.0209080)
Supplement: S2 Table — One-way ANOVA was performed, followed by SNK (Student–Newman–Keuls, p < 0.05) post hoc test. Means indicated with the same letter are not significantly different. (DOCX) [file pone.0209080.s002.docx]

| **Samples** | **Total Ubiquitin** | **Free Ubiquitin** |
| --- | --- | --- |
| **Developmental stages** | | |
| E0-3 | efg | de |
| E8-11 | fg | de |
| E16-19 | efg | e |
| L1 | a | b |
| L2 | cd | c |
| eL3 | fg | de |
| vL3 | de | de |
| P1 | efg | cd |
| P4 | c | c |
| P15 | fg | de |
| male0 | ef | c |
| male3 | g | de |
| female0 | b | b |
| female3 | c | a |
| **Tissues** | | |
| L3 brain | bc | b |
| L3 fatbody | bc | bc |
| salivary gland | c | c |
| testis | b | b |
| ovary | a | a |
| male head | bc | bc |
| male body | bc | bc |
| female head | bc | bc |
| female body | a | a |
