# Supplementary material for: Developmental and tissue specific changes of ubiquitin forms in Drosophila melanogaster
Source: PLoS One. 2018 Dec 13;13(12):e0209080. doi: 10.1371/journal.pone.0209080 (PMC6292614; doi:10.1371/journal.pone.0209080)
Supplement: S3 Table — (DOCX) [file pone.0209080.s003.docx]

| **Buffer Composition** | **Mouse** | | **Drosophila** | |
| --- | --- | --- | --- | --- |
|  | **Buffer F** | **Buffer T** | **Buffer F** | **Buffer T** |
| Tris | 100 mM | | 100 mM | |
| NaCl | 20 mM | | 150 mM | |
| EDTA | --- | | 1 mM | |
| IAA | 10 mM | --- | --- | |
| NEM | --- | | 10 mM | --- |
| MG132 | 20 µM | | 20 µM | |
| DTT | --- | 2 mM | --- | 2 mM |
| CPIC | 1x | | 1x | |
| **pH** | 7.8 | | 7.6 | |
| **Incubation time at RT** | 2 h | | 3 h | |
| **Incubation temperature** | 37 °C | | 25 °C | |

EDTA= Ethylenediaminetetraacetic acid; IAA= Iodoacetamide; NEM= N-Ethylmaleimide; DTT= Dithiothreitol and CPIC= Complete Protease Inhibitor Cocktail
