# Supplementary material for: Developmental and tissue specific changes of ubiquitin forms in Drosophila melanogaster
Source: PLoS One. 2018 Dec 13;13(12):e0209080. doi: 10.1371/journal.pone.0209080 (PMC6292614; doi:10.1371/journal.pone.0209080)
Supplement: S4 Table — After an initial 3-hour incubation, equal aliquots were taken in every 30 minutes that were subjected to SDS-PAGE followed by Western blotting. Relative ubiquitin content of the samples was determined by densitometric analysis of the immunoblots, and their Ub content was compared to the 3h samples in four independent experiments. (DOCX) [file pone.0209080.s004.docx]

| **Relative quantity of the ubiquitin (compared to 3 h sample)** | | | | | |
| --- | --- | --- | --- | --- | --- |
| **Samples** | **3h** | **3.5h** | **4h** | **4.5h** | **5h** |
| **1** | 1.00 | 1.01 | 1.09 | 0.99 | - |
| **2** | 1.00 | 1.03 | 1.04 | 1.05 | 1.14 |
| **3** | 1.00 | 1.12 | 1.18 | 1.05 | 1.11 |
| **4** | 1.00 | 1.15 | 1.04 | 0.98 | 1.00 |
| **Average** | 1.00 | 1.08 | 1.09 | 1.02 | 1.08 |
| **SD** | 0.00 | 0.07 | 0.07 | 0.04 | 0.07 |
